# Effect of Whey Protein Changes on Milk Flavor and Sensory Characteristics During Heating

**DOI:** 10.3390/foods14010033

**Published:** 2024-12-26

**Authors:** Zheting Zhang, Kexin Jiang, Aolin Yang, Kunli Xu, Fanyu Meng, Fang Zhong, Bei Wang

**Affiliations:** 1 School of Food and Health, Beijing Technology and Business University, Beijing 100048, China; zhangzheting22@163.com (Z.Z.); jiangkexinnn@163.com (K.J.); 2230202152@st.btbu.edu.cn (A.Y.); eliauklily@gmail.com (K.X.); f.meng@btbu.edu.cn (F.M.); 2 Key Laboratory of Geriatric Nutrition and Health, Beijing Technology and Business University, Ministry of Education, Beijing 100048, China; 3 School of Food Science and Technology, Jiangnan University, Wuxi 214122, China; fzhong@jiangnan.edu.cn

**Keywords:** milk heat treatment, whey protein, volatile organic compounds, correlation analysis

## Abstract

The flavor of dairy products crucially affects consumer purchase preference. Although the flavor and sensory perception of milk can be influenced by heat treatment during processing, the exact mechanism remains unclear. Therefore, this study analyzed the whey protein content and structural changes of milk heated at different time and temperature combinations and evaluated the flavor compounds and sensory characteristics of milk. The results showed that higher temperatures changed the secondary milk whey protein structures and gradually increased α-lactalbumin, β-lactoglobulin, and lactoferrin denaturation in the milk. There were differences in sensory characteristics of milk heated at different time and temperature combinations. The correlation analysis indicated that whey protein denaturation was negatively correlated with 1-octen-3-ol (*p* < 0.05) and positively associated with hexanal, isovaleric acid, *γ*-nonalactone, methyl palmitate, and phenol (*p* < 0.01). The changes in the content and secondary structure of whey proteins affected the interaction between flavor compounds and whey protein, which affected the release of flavor compounds. Consequently, the sensory characteristics of milk were influenced. This study provides a theoretical basis for exploring the interaction between whey proteins and flavor compounds.

## 1. Introduction

The quality of milk is crucially affected by volatile organic compounds (VOCs), which impact consumer preference [1]. Heat treatment is an essential process during the production of liquid milk and most dairy products. While it effectively controls microorganisms, reduces enzyme activity, and prolongs the shelf-life of milk, heat treatment also affects its flavor and sensory characteristics [2].

Flavor intensity perception is determined by the release of certain compounds from the food matrix and their effect on the sensory system. However, since the chemical composition and structure of milk are complex, the different affinities for flavor compounds influence flavor compound release and perception [3]. Milk contains various VOCs, including alcohols, aldehydes, acids, esters, ketones, sulfur compounds, and other compounds, which mainly result from milk degradation. For example, lipid oxidation and thermal degradation can produce aldehydes or ketones, while the thermal reactions of amino acid side chains produce H_2_S and other sulfur-containing compounds. Furthermore, heating reducing sugar (lactose) in the presence of amino acids, peptides, or proteins may induce the Maillard reaction, which influences the VOC levels [4]. Current research on the flavor of heat-treated milk mainly focuses on the effect of heat treatment on milk flavor, the generation of off-flavors [2,5], and the influence of the Maillard reaction during processing [6]. However, minimal studies are available regarding the interaction between milk proteins and flavor compounds.

The interaction between proteins and flavor compounds depends on the protein (e.g., β-lactoglobulin (β-LG)) and VOC (e.g., aldehydes) properties, such as protein concentration and conformation and VOC structures [7]. In most cases, the protein and flavor compound interactions, such as hydrophobic interactions, hydrogen bonding, ionic bonding, and van der Waals forces, are reversible and can be used to reduce flavor loss during processing and re-release flavor components during consumption [8,9,10]. Some VOCs can irreversibly bind with protein side chains via covalent interactions, playing a vital role in eliminating off-flavors from foods [11]. Casein (CN) is thermally stable, and both casein micelles and caseinate display unstructured properties, while whey protein denaturation in milk is considered a good indicator of the degree of thermal damage [12,13]. Whey protein is heat-sensitive and accounts for about 18–20% of the total milk protein [14]. When exposed to high temperatures, whey proteins undergo denaturation, coagulation, interaction with casein micelles, and Maillard Browning reactions, which change the chemical and nutritional properties of milk [15]. The degree of change depends on the heat treatment time and temperature. Previous research has found that aldehydes, sulfur molecules (especially thiols), and functional furans form covalent bonds with β-LG via Schiff bases, Michael additions, and disulfide bonding, which could cause flavor loss [16]. The methyl distribution in the pyrazine ring significantly affects its release from bovine serum albumin solution [10]. Therefore, understanding the release behavior of flavor compounds in milk proteins, as well as the strength and nature of the interactions between milk proteins and flavor compounds, is particularly important for improving the overall sensory characteristics and quality of milk.

This study used milk collected from the same farm to assess different heat treatment durations (10 min and 30 min) and temperatures (60 °C, 75 °C, and 100 °C). The whey protein denaturation and secondary structure changes after different heat treatments were examined via sodium dodecyl sulfate–polyacrylamide gel electrophoresis (SDS-PAGE), high-performance liquid chromatography (HPLC), and circular dichroism analysis (CD). Furthermore, solid phase microextraction–arrow–gas chromatography–mass spectrometry (SPME-Arrow-GC-MS) and quantitative descriptive analysis (QDA) were employed to analyze flavor compound generation and sensory changes. Finally, the correlation between the active proteins (α-lactalbumin (α-LA), β-LG, lactoferrin (LF)) and milk flavor compounds was explored. This study provides a reference for clarifying the interaction between the whey proteins and flavor compounds in milk by examining the structural changes of whey proteins; this is highly significant for improving the properties of milk and developing rational design strategies for sterilization processes during industrial production.

## 2. Materials and Methods

### 2.1. Materials

Chromatographic-grade hexane and acetonitrile were purchased from Energy Chemical Co., Ltd. (Shanghai, China). The n-alkane standards (C7~C30) were supplied by o2si Smart Solutions (Charleston, SC, USA), while the chromatographic-grade 2-methyl-3-heptanone was obtained from Sigma-Aldrich (Shanghai, China). Acetic acid and hydrochloric acid were of analytical grade, purchased from Beijing Mreda Technology Co., Ltd. (Beijing, China). The chromatographic-grade trifluoroacetic acid was purchased from Shanghai Aladdin Biochemical Technology Co., Ltd. (Shanghai, China). Shanghai Yuanye Bio-Technology Co., Ltd. (Shanghai, China) supplied the α-LA (≥85%), while β-LG (≥90%) and LF (≥98%) were purchased from Sigma-Aldric (Shanghai, China). Coomassie Brilliant Blue R-250 was obtained from Bio-Rad Laboratories (Hercules, CA, USA). Beijing Solarbio Science and Technology Co., Ltd. (Beijing, China) supplied the Rainbow 180 broad spectrum protein marker (11–180 KD), while the 5× protein sampling buffer (without DTT) and 2× protein sampling buffer (with DTT) were purchased from Beijing BioDee BioTech Co., Ltd. (Beijing, China).

### 2.2. Sample Collection and Preparation

The raw bovine milk sample was obtained from the bulk tank at Junlebao Dairy Co., Ltd. (Shijiazhuang, Hebei, China) and then transported to the laboratory within a 12-h period via a 4 °C cold chain. It was then mixed with 0.05% NaN_3_ to prevent microbial growth, and labeled as raw [17]. The raw milk was subsequently heated in a water bath (SHJ-1AB, Jintan Liangyou Instrument Co., Ltd., Changzhou, Jiangsu, China) at different heating times and temperatures, which included 60 °C for 10 min, 60 °C for 30 min, 75 °C for 10 min, 75 °C for 30 min, 100 °C for 10 min, and 100 °C for 30 min, and immediately cooled in a 0 °C ice water bath for 30 min. The heated samples were labeled 6010, 6030, 7510, 7530, 10010, and 10030, respectively. All seven samples were obtained from the same milk source and stored in a refrigerator at −20 °C for further analysis.

The whey protein was extracted using a method delineated by Qi et al. [18] with slight modifications. Seven different heat-treated milk samples were immediately defatted via centrifugation at 5000× *g* for 30 min at 25 °C using a bench-top refrigerated centrifuge (Heraeus Multifuge ×1R, Thermo Fisher Scientific, Cleveland, OH, USA). The samples were acidified to pH 4.6 using 1 N acetic acid or hydrochloric acid, respectively, kept at room temperature (20–22 °C) for 10 min, and centrifuged at 10,000× *g* for 25 min at 4 °C using a bench-top refrigerated centrifuge (Heraeus Multifuge ×1R, Thermo Fisher Scientific, Cleveland, OH, USA). The top residual fat layer and casein precipitate were discarded, and centrifugation was repeated at 10,000× *g* for 25 min at 4 °C to ensure complete separation of the soluble whey, casein, and fat. The soluble whey was dialyzed to minimize or eliminate the interference of water-soluble vitamins in subsequent experiments. Finally, all the whey protein samples were passed through 0.45 μm filter membranes and stored in a refrigerator at 4 °C until analysis.

### 2.3. Protein Analysis

#### 2.3.1. Determination of the Protein Profile

The protein profile was determined using SDS-PAGE based on the method described by Zenker et al. [19], with slight modifications. A 15 μL milk sample was mixed with 65 μL ultrapure water, after which, 80 μL of 2× loading buffer (with and without DTT) was added, respectively. The samples were placed in a boiling water bath for 10 min, followed by rapid cooling in an ice water bath. Next, 5 μL of the prepared protein sample was placed on a 4–20% Super-PAGETM Bis-Tris gel (Epizyme, Shanghai, China) and separated using a Mini P-4 vertical electrophoresis device (Cavoy, Beijing, China). The sample was stained with Coomassie Brilliant Blue R-250 (0.01%, *w*/*v*) for 1 h, decolorized with a decolorizing solution, and shaken overnight. The electrophoresis gels were photographed using a UVP GDS-8000 image analysis system (UVP, Inc., Upland, CA, USA).

#### 2.3.2. Determination of Denaturation Rate of Active Protein

An HPLC system equipped with an AdvanceBio RP-mAb C4 column (4.6 × 50 mm, 3.5 µm) and a diode array detector (Agilent 1260, Agilent Technologies Co., Santa Clara, CA, USA) was used to quantify the α-LA, β-LG, and LF in whey protein samples [20]. Gradient elution was performed by mixing different ratios of mobile phase A (aqueous phase consisting of 0.1% trifluoroacetic acid) and mobile phase B (acetonitrile comprising 0.1% trifluoroacetic acid) at different times. The mobile phase was applied at a constant flow rate of 1 mL/min and a sample injection volume of 10 μL. The α-LA and β-LG levels were determined at a column temperature of 60 °C and a detection wavelength of 210 nm. The gradient program started with 30% B, which was increased to 55% B at 10 min and 70% B at 10.1 min, where it was maintained until 12 min. Then, it was decreased to the initial 30% B at 12.1 min, where it was maintained until the end of the run at 17 min. The LF level was determined at a column temperature of 70 °C and a detection wavelength of 280 nm. The gradient program started with 5% B for 2 min, which increased to 80% B at 5 min, where it was maintained until 8 min, after which, it was reduced to the initial 5% B at 9 min and maintained until the end of the run at 13 min. Five concentrations of each compound were used for calibration.

The active protein measurements were performed using a method described by Rynne et al. [21] with some modifications. The results were expressed as a percentage of active protein denatured (*APD*) after heating. The following equation was used for calculation:

(1) $$APD(\%)=100\times \frac{{AP}_{rm}-{AP}_{hm}}{{AP}_{rm}}$$

where *AP_rm_* and *AP_hm_* represent the active protein contents in the raw and heated milk samples, respectively.

#### 2.3.3. Determination of Secondary Structure of Whey Protein

A JASCO J-1500 Circular Dichroism Spectrophotometer (Jasco, Tokyo, Japan) was used to analyze the secondary structures of the sample proteins. The whey protein solution acidified using acetic acid was employed for the CD experiment to avoid the influence of chloride ions [22]. Here, 0.1 mg/mL whey protein samples were placed in a 1 mm quartz colorimetric dish, and the spectrum in the far-ultraviolet region (190–260 nm) was measured. The results were analyzed via the software CDNN (v.2.1, Applied Photophysics Ltd., Leatherhead, UK).

### 2.4. Analysis and Identification of the VOCs

#### 2.4.1. Extraction of VOCs in Milk Samples

The VOCs in milk samples were extracted using Smart SPME-Arrow extraction fiber (DVB/Carbon WR/PDMS (divinylbenzene, carbon wide range, polydimethylsiloxane), 1.10 mm outer diameter, 120 μm stationary-phase thickness). Here, 5 g of the milk sample, 0.5 g of NaCl, and 0.6 μL of 816 mg/kg of a 2-methyl-3-heptanone internal standard solution (n-hexane as solvent) were taken into a 20 mL headspace vial (which has a magnetic cap and a polytetrafluoroethylene/silica gel spacer) and mixed well. After equilibration at 45 °C for 20 min, the SPME-Arrow extraction head was inserted into the vial and adsorbed in the headspace for 30 min.

#### 2.4.2. GC-MS Analysis

The VOCs were identified using a GC-MS-TQ8040 NX system equipped with an AOC-6000 multifunctional injector (Shimadzu, Kyoto, Japan), while an SH-Polar Wax capillary column (60 m × 0.25 mm, 0.25 μm) (Shimadzu, Kyoto, Japan) was used for separation. The adsorbed extraction head was transferred to the GC-MS injection port and desorbed at 250 °C for 1 min. The initial temperature of the chromatography oven was 40 °C, held for 5 min, and then increased to 250 °C at a rate of 3 °C/min, and maintained for 2 min at 250 °C. The total run time was 77 min, and the solvent delay time was 1.82 min. High-purity helium was used as the carrier gas (99.99%) with a flow rate of 1 mL/min. The ionization source was an electron bombardment ionization source of 70 eV, and the temperature of the ion source and interface were 200 and 250 °C, respectively. The data were collected via multiple reaction monitoring mode in a mass scanning range of 33~550 *m*/*z* [23,24].

The standard NIST14 mass spectral library and Shimadzu Flavor Substance databases were used for VOC identification. The relative concentration of each VOC was calculated via internal standard semi-quantification using the following equation:

(2) $$C_{x}=\frac{C_{i}}{A_{i}}\times A_{x}$$

where *C_x_* and *A_x_* represent the relative concentration and chromatographic peak areas of a VOC, and *C_i_* and *A_i_* denote the concentration and chromatographic peak areas of the internal standard (2-methyl-3-heptanone).

### 2.5. Sensory Evaluation

The sensory characteristics of milk samples were evaluated by QDA [25]. The sensory evaluation group consisted of 12 students (two males and 10 females between 18 and 27 years old) recruited from the Beijing Technology and Business University. All participants in the sensory evaluation experiment carefully read the study description before the experiment and understood the subjects, procedure, risks, and benefits of the study. And all signed the informed consent. The panelists attended five 2-h training sessions for basic taste perception and the use of sensory scales. Then, the evaluators established a list of the sensory attributes of the samples via consensus after discussion and identified definitions and references for each attribute (Table S1). The sensory evaluation panel was trained according to the established glossary of sensory descriptions until all panelists were familiar with the terms in the table and the intensity of the attributes.

The sensory evaluation was performed at room temperature (25 ± 1 °C). Here, 10 mL of each of the seven milk samples were placed in disposable sensory evaluation cups and numbered with random three-digit numbers. The samples were scored on a point scale from 0 to 9 points (1 point on a scale of 1), while the final score for each term represented the mean value.

### 2.6. Statistical Analysis

Each experiment was repeated in triplicate for each sample, and the results were expressed as mean ± standard deviation. The experimental data were recorded, and preliminary calculations were performed using Microsoft Excel 2021 (Microsoft, Redmond, WA, USA). One-way ANOVA was performed using the SPSS 27.0 software (SPSS Inc., Chicago, IL, USA) to assess the differences between the samples (*p* < 0.05). Origin 2021 (Origin Lab Corporation, Northampton, MA, USA) was used to create bar graphs, line graphs, radar plots, and correlation plots. PCA was performed using the 14.1.0 SIMCA software (Umetrics AB, Umeå, Sweden). ChiPlot (https://www.chiplot.online/, accessed on 6 September 2024) was used for heatmap and advanced cluster analysis, while XLSTAT software (version 2019.2.2, Addinsoft, New York, NY, USA) was employed for partial least squares regression (PLSR).

## 3. Results and Discussion

### 3.1. Protein Profile and Structural Changes

#### 3.1.1. Protein Denaturation and Aggregation

**Figure 1:**
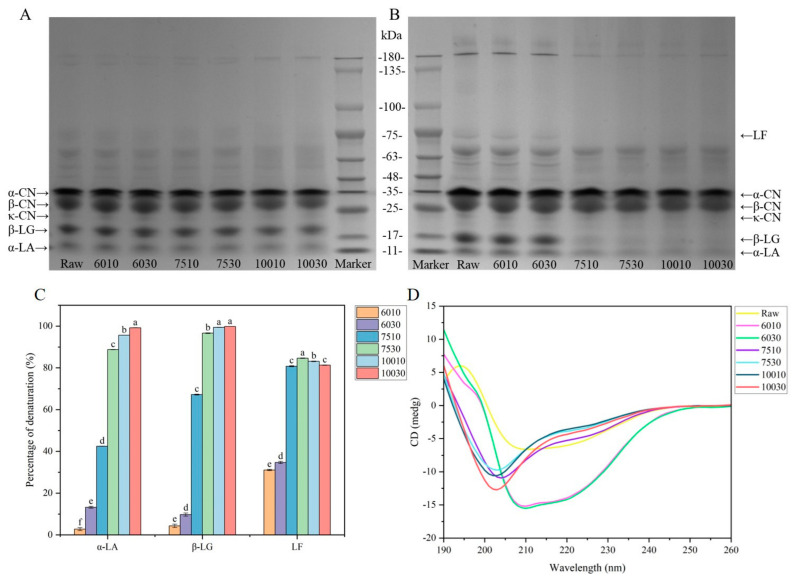
Changes in the milk proteins after different heat treatments. (**A**) The SDS-PAGE profile in reducing conditions. (**B**) The SDS-PAGE profile in non-reducing conditions. (**C**) The denaturation rates of the active proteins (α-LA, β-LG, and LF). (**D**) The CD analysis of the whey protein solution. ^a–f^ According to the Tukey test, different superscripts of the same active protein indicate significant differences (*p* < 0.05).

SDS-PAGE is increasingly applied in milk protein studies due to its high resolution and quantitative results [26]. Reducing and non-reducing SDS-PAGE were performed to determine the protein composition, aggregate formation, and protein denaturation of milk after different heat treatments. Figure 1A,B show the electrophoretic profiles of the protein solutions during SDS-PAGE after different heat treatments. No significant changes were evident in the α-CN and β-CN bands as the treatment temperatures increased. Contrarily, β-LG and α-LA, the main components in milk whey proteins, are thermally unstable due to their significant number of secondary and tertiary structures [27]. The denaturation of these components started at 65 °C and mainly occurred when the milk was heated above 80 °C [14]. When the temperature increased to 75 °C and 100 °C, the β-LG and α-LA bands became lighter and more obvious in the non-reducing state. The non-reducing SDS-PAGE protein bands were less obvious than those in reducing conditions, indicating that heat-induced protein aggregation occurred mainly via covalent bonding. This caused protein aggregates that were difficult to separate [28,29]. In reducing conditions, the β-LG displayed no significant changes when heated at 60 °C for 10 min, 60 °C for 30 min, and 75 °C for 10 min (Figure 1A). Since β-LG was in a “molten globule state” at this time, the conformational change was reversible [30]. Figure 1B shows the electrophoretic profile in non-reducing conditions. The β-LG, α-LA, and LF bands disappeared gradually as the heating temperature increased to 75 °C and 100 °C, which was consistent with the results of previous research [31]. This differed significantly from the results in Figure 1A, possibly because heating caused protein unfolding to expose the thiol groups. This caused protein aggregation and complex formation between α-LA and β-LG, α-LA and κ-CN, and β-LG and κ-CN via thiol-disulfide exchange reactions [14,32]. Furthermore, due to higher heat sensitivity, the β-LG bands were lighter than those of α-LA [27]. This study primarily examined whey proteins in subsequent experiments due to their sensitivity to heat treatment and tendency to decrease in content at higher temperatures.

#### 3.1.2. Denaturation Rate of the Active Milk Proteins

HPLC was used to analyze the milk protein components due to its high resolution, accurate, and repeatable results [33]. The whey protein profile showed that β-LG (50%) and α-LA (20%) represented the primary whey proteins in the milk, with a small amount of LF (4%) [34]. Figure 1C shows the denaturation percentage of β-LG, α-LA, and LF after different heat treatments. Heat treatment at temperatures above 60 °C caused whey protein denaturation, mainly β-LG and α-LA [34]. At 60 °C, the LF denaturation degree exceeded that of β-LG and α-LA, indicating that it displayed higher thermal sensitivity. This was because the LF contained 17 intramolecular disulfide bonds and no free thiol groups, causing thermal denaturation. Moreover, LF exhibited two denaturation temperatures at a neutral pH: 60.4 °C for lobe N and 89.1 °C for lobe C. The temperature sensitivity of the two lobes differed since the C-lobe is denser than the N-lobe [35]. A heat treatment temperature of 75 °C increased the thermal denaturation percentages of the three whey proteins, with almost complete denaturation evident at 100 °C.

#### 3.1.3. Secondary Structures of the Whey Proteins

α-LA and β-LG are globular proteins that naturally fold into unique, compact, highly ordered structures. Regular conformational regions, known as α-helix and β-folds, are interconnected to form a 3D structure [36,37]. Proteins undergo structural alterations during processes such as ligand binding. Figure 1D shows the changes in the secondary whey protein structures after different heat treatments. The CD band of the protein in the far ultraviolet region (190–260 nm) was mainly derived from the amides of the protein backbone and was sensitive to their conformations [18].

The α-helix displayed a positive peak at 190 nm and a negative peak at 208 nm in the CD spectrum due to π_nb_ π* transition exciton splitting, while another negative peak was evident at 220 nm due to nπ* transition [38]. The β-sheet exhibited a positive peak near 195 nm and a negative peak near 215 nm, while the β-turn displayed a maximum peak at 200 nm and a minimum peak at 220 nm. Furthermore, the proteins without dominant secondary structures (random coil) presented a negative peak near 200 nm [38,39]. Thermal denaturation of milk typically involves protein molecule unfolding and the irreversible aggregation of unfolded molecules [40]. As shown in Figure 1D, the raw sample displayed a β-sheet structure. Compared with the raw samples, the maximum and minimum values of the CD spectra at 195 nm and 215 nm of 6010 and 6030 blue-shifted to 190 nm and 208 nm, respectively, while the negative ellipticity gradually increased between 208 and 230 nm. The peak shifts indicated protein β-sheet loss, while the α-helix and random coil content increased, indicating that the whey protein changed from an ordered to a disordered structure after heat treatment [41]. The CD spectra of the whey proteins were similar in the milk heated at 75 °C and 100 °C, with a significant negative peak near 200 nm, indicating an increase in the random coil content. The CD spectra results indicated that the different heat treatments altered the secondary structures of the milk whey proteins. Enhanced hydration and partial tertiary structure loss were evident at 50 °C. The mobility of certain polypeptide chain sections increased at a pasteurization temperature between 65 °C and 71 °C, revealing secondary structures such as α-helix and β-sheet. Temperatures between 70 °C to 80 °C caused disulfide bond breakage, tertiary structure loss, and partial secondary structure loss. Disulfide bond breakage and secondary structure loss were evident between 80 °C and 90 °C, while temperatures between 90 °C and 100 °C caused intermolecular disulfide bond formation and hydrophobic interactions. Protein coagulation, lysine formation, and Maillard reactions were apparent between 100 °C and 105 °C [42].

Table 1 shows the percentages of the whey protein structural changes after different heat treatments, with the α-helix in the raw sample at 15.5%, the β-sheet at 34.6%, and the β-turn at 19.6%. When heated at 60 °C, the proportion of α-helix and random coil increased, while the β-sheet decreased. Furthermore, a higher temperature and extended time increased the random coil percentage to 31.8% in 7510 and 32.0% in 10010, and the β-turn increased, which was consistent with the results of previous research [18]. In summary, higher treatment temperatures altered the secondary structure and interaction forces of the whey proteins, which affected the milk flavor.

### 3.2. VOCs Characteristics of Different Heat-Treated Milk

The VOCs in dairy products typically include alcohols, aldehydes, acids, esters, and ketones, which contribute significantly to the overall aroma. HS-SPME-arrow-GC-MS was used for the qualitative and quantitative analysis of the VOCs in the milk exposed to different heat treatments (Table S2). A total of 59 VOCs were identified, including nine alcohols, six aldehydes, fifteen acids, eleven esters, ten ketones, and eight other compounds. The total VOC content ranged between 112.39 μg/kg and 595.24 μg/kg.

Alcohols originate via various metabolic pathways and typically produce floral, fruity, and grassy flavors in dairy products [43]. A total of nine alcohols were identified in the milk, with high levels of 1-butanol, 2-methyl-1-butanol, 1-hexanol, and 2-ethyl-1-hexanol. Aldehydes, such as hexanal and octanal, provided the milk samples with grassy and fatty flavors and increased at higher temperatures. (*E*)-2-nonenal presented a cucumber-like flavor and was higher after heat treatment at 75 °C and 100 °C. Acids, especially short- and medium-chain fatty acids, contribute significantly to the aroma of dairy products due to their low perceived thresholds. For example, acetic acid, propanoic acid, and butyric acid present a strong sour and putrid flavor [44]. However, long-chain fatty acids play a minor role in flavor due to their higher thresholds [44]. Although free fatty acids contribute to the aroma of dairy products, excessive amounts can cause a rancid flavor [44]. Not only are fatty acids aromatic compounds, but they are also precursors of other flavor compounds such as alcohols, aldehydes, ketones, and lactones [44]. Esters usually present pleasant flavors, such as sweet, peachy, and coconut aromas. Ketones are mainly present in the form of methyl ketones, which are free fatty acid derivatives. The free fatty acids are first oxidized to β-keto acids and then form the corresponding methyl ketones [44]. Furthermore, a strong correlation is evident between the methyl ketone concentration in milk samples and various heat indicators such as furosine, lactulose, and undenatured whey protein [45]. Ketones have low perception thresholds and contain 2-heptanone and 2-nonanone, which present soapy and hot milk flavors in dairy products [46]. Sulfur compounds are commonly associated with the cooking flavor of milk and significantly affect its flavor [43]. Two sulfur compounds, namely dimethyl sulfoxide and dimethyl sulfone, were found in the seven milk samples, and dimethyl sulfone presents unpleasant sulfur and burnt flavors. Dimethyl sulfide oxidation results in intermediate dimethyl sulfoxide formation [47].

**Figure 2:**
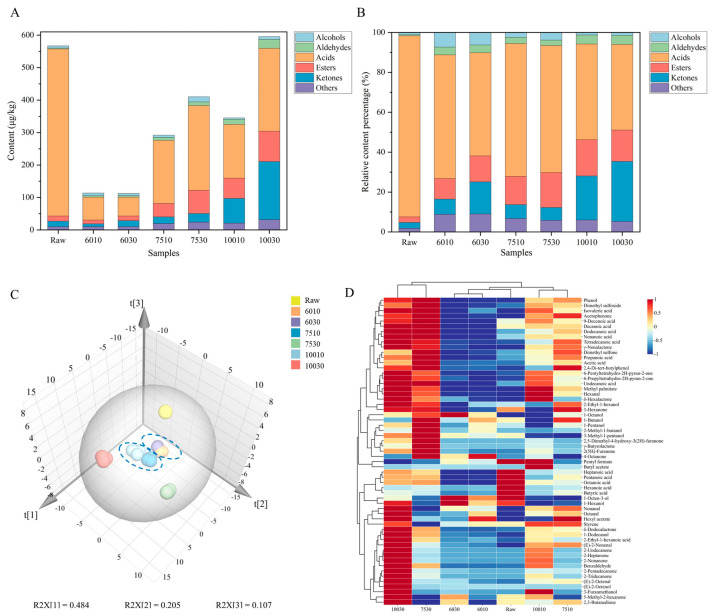
The VOCs identified via HS-SPME-Arrow-GC-MS in the milk after different heat treatments. (**A**) The relative VOC content. (**B**) The relative VOC content percentage. (**C**) The PCA analysis of the VOCs. (**D**) The clustering heat map analysis of the VOCs.

Stacked histograms were created to visualize the level of each compound in the samples (Figure 2A,B). The raw samples contained the highest acid level at 514.52 μg/kg, with a relative content of 91%. This was consistent with the findings of Yuan et al. [5], who found that the acid content in raw milk was significantly higher than in milk treated at 65 °C and 135 °C for 30 min. Furthermore, the total VOC content in the milk samples increased gradually at higher temperatures, as shown by 6010 ≈ 6030 < 7510 < 10010 < 7530 < 10030 (Figure 2A). At the same heating temperature, the total VOC content increased as the heating time was extended.

Principal component analysis (PCA) was used to compare the differences between the VOCs in the seven milk samples (Figure 2C). The results showed a cumulative variance contribution of 79.6%, indicating that the 3D PC covered most of the odor information of all the samples. The raw, 7530, and 10030 groups were clustered into one category. Additionally, 6010 and 6030 were clustered into one group, while 7510 and 10010 were grouped into another, indicating that their overall flavors were similar.

A cluster heat map was used to illustrate the classification and comparability of the flavor compounds produced in the milk exposed to different heat treatments (Figure 2D). The raw group displayed a relatively high acid content compared to other samples. The VOC levels in 6010 and 6030 were fairly low and clustered into one group, which was consistent with the PCA results. The VOC content in 10010 and 7510 was higher than in 6010 and 6030 but lower than milk samples heated at the same temperature for 30 min. The 10030 and 7530 milk samples displayed high VOC levels and were grouped together.

### 3.3. Sensory Characteristics

**Figure 3:**
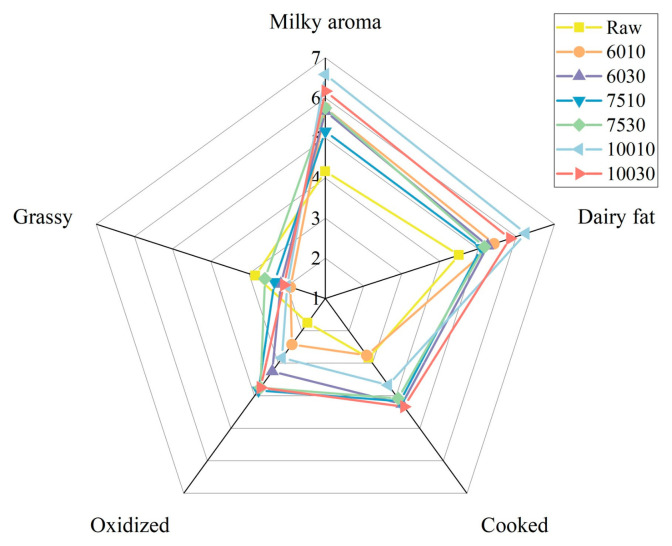
The sensory evaluation scores of the milk exposed to different heat treatments.

The milky, dairy fat, cooked, oxidized, and grassy aromas of the samples were evaluated to assess the overall milk flavor variation after different heat treatments. Figure 3 shows the flavor profiles of the milk samples exposed to seven heat treatment conditions, indicating the presence of significant differences.

Table 2 presents the results of the QDA score analysis of the milk samples exposed to different heat treatments. The 10010 samples displayed the highest milky aroma score of 6.58, followed by 10030 at 6.17. The diary fat flavor score of 10010 was also the highest of the seven samples at 6.25. The aldehydes and ketones resulting from fat oxidation at higher temperatures increased the oxidized milk flavor score [48]. The cooked flavor resulted from whey protein denaturation, which exposed the sulfhydryl groups of dimethyl sulfide and the subsequent sulfides [49]. The raw sample displayed the highest grassy flavor score at 2.83, while different feeds were primarily responsible for the flavor variation between the raw milk specimens [50]. A survey showed that fresh milk obtained from silage-based and by-product-based feeding systems exhibited fatty acid variation [51]. In addition, many VOCs in milk, such as terpenes, esters, aldehydes, ketones, and alcohols, are related to feed ingredients [50]. However, studies have shown that VOCs are more significantly affected by heat treatment than by feed [52].

### 3.4. Correlation Analysis

**Figure 4:**
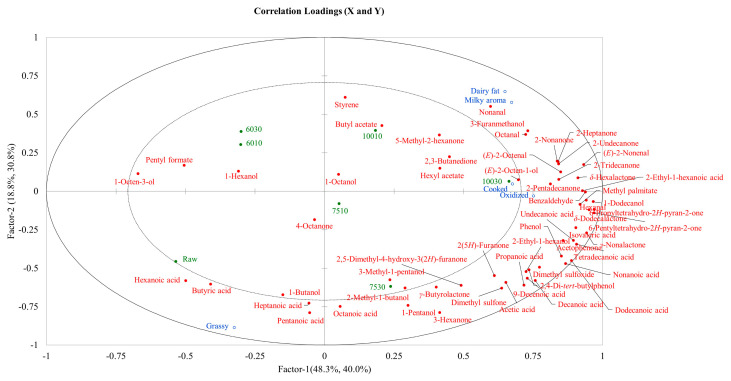
The PLSR results of the sensory evaluation and VOCs of milk under different heat treatment conditions.

PLSR was used to assess the correlations between the sensory attributes and VOCs (Figure 4). Most flavor compounds are located on the positive axis and are positively correlated with milky, dairy fat, cooked, and oxidized aromas. However, higher temperatures increased the types and levels of flavor compounds in the milk samples. The raw sample contained high levels of hexanoic acid, butyric acid, heptanoic acid, and other acid compounds. Both the raw sample and grassy flavor were located in the third quadrant. This indicated that the raw samples exhibited a stronger grassy taste, which was consistent with the sensory evaluation score. Similarly, 10030 and 10010 were located in the first quadrant, indicating that both displayed strong milky, dairy fat, and cooked aromas, which was also consistent with the sensory evaluation results.

**Figure 5:**
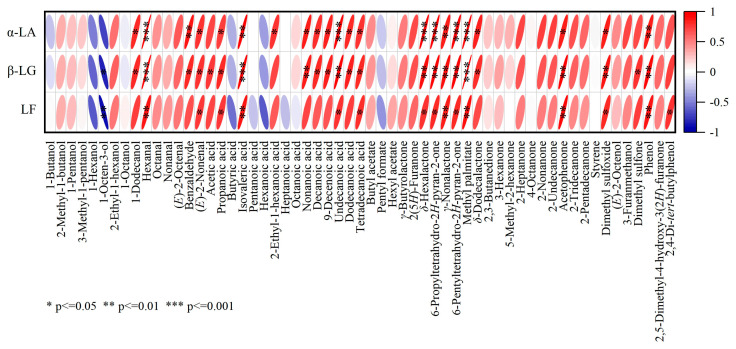
The correlation plot of the active proteins and VOCs in the milk exposed to different heat treatments.

A correlation plot was created to investigate the association between whey protein denaturation and flavor compounds (Figure 5). The results showed that both α-LA and β-LG were negatively correlated with 1-butanol, 1-hexanol, 1-octen-3-ol, 1-octanol, butyric acid, hexanoic acid, pentyl formate, and positively associated with other compounds. The strongest negative correlation was evident between β-LG and 1-octen-3-ol (*p* ≤ 0.05). In addition to these compounds, LF was negatively correlated with pentanoic, heptanoic, and octanoic acid, showing the strongest negative correlation with 1-octen-3-ol (*p* ≤ 0.01). A strong positive correlation was evident between α-LA and hexanal, undecanoic acid, *δ*-hexalactone, 6-propyltetrahydro-2*H*-pyran-2-one, 6-pentyltetrahydro-2*H*-pyran-2-one, and methyl palmitate (*p* ≤ 0.001), while β-LG showed the strongest positive association (*p* ≤ 0.001) with hexanal, isovaleric acid, and methyl palmitate. Overall, the three active proteins were positively associated with hexanal, isovaleric acid, *γ*-nonalactone, methyl palmitate, and phenol (*p* ≤ 0.01). The relationship between whey protein denaturation and these flavor compounds can be further explored in future studies.

To explore the correlation between milk flavor compounds and whey protein denaturation as comprehensively as possible, this study used whole milk to produce a significant variety of VOCs. However, due to the complex composition of milk, the fat and lactose it contains may also react or interact with flavor compounds. Subsequent experiments can further explore the effects of fat and lactose on flavor compounds. And experiments can also be conducted with whey protein monomers and compound standards to further explore the correlation between milk whey protein and VOCs, while new techniques can be employed for more comprehensive analysis. For example, molecular docking techniques and molecular dynamics simulations are used to predict the binding mode and affinity of samples by simulating the forces and sites of interaction between proteins and flavor compounds [53].

## 4. Conclusions

Milk is commonly heat-treated during industrial processing to decrease microorganism and enzyme activity, consequently extending its shelf life and stability and reducing the possibility of deterioration. Whey protein is heat-sensitive, making it susceptible to irreversible degradation due to structural alterations, subsequently impacting the flavor production and release. Analysis revealed that the degree of β-LG, α-LA, and LF denaturation increased after heat treatment. Changes are also evident in the secondary whey protein structures. In addition to the substantial number of acid compounds in the raw milk, the content of other flavor compounds also increases as heat treatment intensifies. The acid compounds further convert into other flavor compounds. Higher temperatures increase the milky aroma and fat flavor scores, with that of the raw milk grassy flavor being the highest. Correlation analysis showed that the active proteins display a strong negative correlation with 1-octen-3-ol (*p* < 0.05) and a significant positive association with compounds such as hexanal and methyl palmitate (*p* < 0.01), providing a reference for subsequent studies on the effect of whey protein denaturation on flavor. However, the binding sites and interactions between whey proteins and the flavor compounds in milk require further clarification in subsequent research.

## Figures and Tables

**Table 1:** Changes in the structural whey protein content after different heat treatments.

Sample	α-Helix (%)	β-Sheet (%)	β-Turn (%)	Random Coil (%)
Raw	15.5 ± 0.08 ^c^	34.6 ± 0.08 ^a^	19.6 ± 0.00 ^g^	30.3 ± 0.08 ^e^
6010	25.1 ± 0.05 ^b^	8.4 ± 0.05 ^e^	21.4 ± 0.12 ^e^	45.1 ± 0.21 ^a^
6030	27.2 ± 0.05 ^a^	8.3 ± 0.05 ^e^	20.5 ± 0.00 ^f^	43.9 ± 0.05 ^b^
7510	12.5 ± 0.05 ^d^	30.4 ± 0.19 ^d^	25.3 ± 0.05 ^b^	31.8 ± 0.19 ^c^
7530	9.8 ± 0.05 ^f^	34.8 ± 0.05 ^a^	24.0 ± 0.05 ^d^	31.3 ± 0.00 ^d^
10010	9.4 ± 0.05 ^g^	33.6 ± 0.12 ^b^	25.0 ± 0.08 ^c^	32.0 ± 0.08 ^c^
10030	11.0 ± 0.05 ^e^	32.1 ± 0.29 ^c^	26.3 ± 0.09 ^a^	30.6 ± 0.26 ^e^

^a–g^ According to the Tukey test, different superscripts in the same column indicate significant differences (*p* < 0.05).

**Table 2:** The QDA scores of the milk exposed to different heat treatments.

Descriptors	Raw	6010	6030	7510	7530	10010	10030
Milky aroma	4.17 ^d^	5.75 ^b^	5.67 ^bc^	5.17 ^c^	5.75 ^b^	6.58 ^a^	6.17 ^ab^
Dairy fat	4.50 ^d^	5.42 ^bc^	5.25 ^bc^	5.08 ^c^	5.17 ^c^	6.25 ^a^	5.83 ^ab^
Cooked	2.83 ^c^	2.75 ^c^	4.25 ^a^	4.17 ^a^	4.08 ^a^	3.67 ^b^	4.33 ^a^
Oxidized	1.75 ^e^	2.42 ^d^	3.25 ^bc^	3.83 ^a^	3.75 ^ab^	2.83 ^cd^	3.75 ^ab^
Grassy	2.83 ^a^	1.92 ^c^	2.17 ^bc^	2.33 ^bc^	2.58 ^ab^	2.00 ^c^	2.08 ^c^

^a–e^ According to the Tukey test, different superscripts in the same row indicate significant differences (*p* < 0.05).

## Data Availability

The original contributions presented in this study are included in the article/Supplementary Materials; further inquiries can be directed to the corresponding author.

## Supplementary Materials

The following supporting information can be downloaded at https://www.mdpi.com/article/10.3390/foods14010033/s1, Table S1: The list of milk attributes in the Quantitative Description Analysis; Table S2: The identification and quantification of the VOCs in the milk exposed to different heat treatments using HS-SPME-Arrow-GC-MS.

## References

[1] Shao Y., Liu X., Zhang Z., Wang P., Li K., Li C. (2023). Comparison and discrimination of the terpenoids in 48 species of huajiao according to variety and geographical origin by E-nose coupled with HS-SPME-GC-MS. Food Res. Int..

[2] Coolbear T., Janin N., Traill R., Shingleton R. (2022). Heat-induced changes in the sensory properties of milk. Int. Dairy J..

[3] Wang K., Arntfield S.D. (2017). Effect of protein-flavour binding on flavour delivery and protein functional properties: A special emphasis on plant-based proteins. Flavour. Fragr. J..

[4] Cadwallader K.R., Singh T.K., McSweeney P., Fox P.F. (2009). Flavours and Off-Flavours in Milk and Dairy Products. Advanced Dairy Chemistry: Volume 3: Lactose, Water, Salts and Minor Constituents.

[5] Yuan N., Chi X., Ye Q., Liu H., Zheng N. (2023). Analysis of Volatile Organic Compounds in Milk during Heat Treatment Based on E-Nose, E-Tongue and HS-SPME-GC-MS. Foods.

[6] Zhang Y., Yi S., Lu J., Pang X., Xu X., Lv J., Zhang S. (2021). Effect of different heat treatments on the Maillard reaction products, volatile compounds and glycation level of milk. Int. Dairy J..

[7] Kühn J., Considine T., Singh H. (2008). Binding of Flavor Compounds and Whey Protein Isolate as Affected by Heat and High Pressure Treatments. J. Agric. Food Chem..

[8] Heng L., van Koningsveld G.A., Gruppen H., van Boekel M.A.J.S., Vincken J.P., Roozen J.P., Voragen A.G.J. (2004). Protein–flavour interactions in relation to development of novel protein foods. Trends Food Sci. Technol..

[9] Zhang J., Kang D., Zhang W., Lorenzo J.M. (2021). Recent advantage of interactions of protein-flavor in foods: Perspective of theoretical models, protein properties and extrinsic factors. Trends Food Sci. Technol..

[10] Ma Y.-J., Wu J.-H., Li X., Xu X.-B., Wang Z.-Y., Wu C., Du M., Song L. (2019). Effect of alkyl distribution in pyrazine on pyrazine flavor release in bovine serum albumin solution. RSC Adv..

[11] Gu S., Dai W., Chong Y., Lyu F., Zhou X., Ding Y. (2020). The binding of key fishy off-flavor compounds to silver carp proteins: A thermodynamic analysis. RSC Adv..

[12] Lin S., Sun J., Cao D., Cao J., Jiang W. (2010). Distinction of different heat-treated bovine milks by native-PAGE fingerprinting of their whey proteins. Food Chem..

[13] Daniloski D., McCarthy N.A., Vasiljevic T. (2022). Impact of heating on the properties of A1/A1, A1/A2, and A2/A2 β-casein milk phenotypes. Food Hydrocoll..

[14] Jovanovic S., Barac M., Macej O., Vucic T., Lacnjevac C. (2007). SDS-PAGE Analysis of Soluble Proteins in Reconstituted Milk Exposed to Different Heat Treatments. Sensors.

[15] Maćej O., Jovanović S., Denin-Djurdjević J.D. (2002). The influence of high temperatures on milk proteins. Hem. Ind..

[16] Anantharamkrishnan V., Hoye T., Reineccius G.A. (2020). Covalent Adduct Formation Between Flavor Compounds of Various Functional Group Classes and the Model Protein β-Lactoglobulin. J. Agric. Food Chem..

[17] On-Nom N., Grandison A.S., Lewis M.J. (2010). Measurement of ionic calcium, pH, and soluble divalent cations in milk at high temperature. J. Dairy Sci..

[18] Qi P.X., Ren D., Xiao Y., Tomasula P.M. (2015). Effect of homogenization and pasteurization on the structure and stability of whey protein in milk1. J. Dairy Sci..

[19] Zenker H.E., Raupbach J., Boeren S., Wichers H.J., Hettinga K.A. (2020). The effect of low vs. high temperature dry heating on solubility and digestibility of cow’s milk protein. Food Hydrocoll..

[20] Bordin G., Cordeiro Raposo F., de la Calle B., Rodriguez A.R. (2001). Identification and quantification of major bovine milk proteins by liquid chromatography. J. Chromatogr. A.

[21] Rynne N.M., Beresford T.P., Kelly A.L., Guinee T.P. (2004). Effect of milk pasteurization temperature and in situ whey protein denaturation on the composition, texture and heat-induced functionality of half-fat Cheddar cheese. Int. Dairy J..

[22] Kelly S.M., Price N.C. (2000). The use of circular dichroism in the investigation of protein structure and function. Curr. Protein Pept. Sci..

[23] Han H., Zhang Z., Yang Z., Blank I., Zhong F., Wang B., Wang Y., Zeng H. (2024). A comparative study to determine the key aroma components of yogurt aroma types based on Sensomics and Flavoromics. Food Chem..

[24] Kessler J.C., Vieira V., Martins I.M., Manrique Y.A., Ferreira P., Calhelha R.C., Afonso A., Barros L., Rodrigues A.E., Dias M.M. (2023). The potential of almonds, hazelnuts, and walnuts SFE-CO2 extracts as sources of bread flavouring ingredients. Food Chem..

[25] Silva H.L.A., Balthazar C.F., Silva R., Vieira A.H., Costa R.G.B., Esmerino E.A., Freitas M.Q., Cruz A.G. (2018). Sodium reduction and flavor enhancer addition in probiotic prato cheese: Contributions of quantitative descriptive analysis and temporal dominance of sensations for sensory profiling. J. Dairy Sci..

[26] Jin Y.K., Park Y.W. (1996). SDS-PAGE of Proteins in Goat Milk Cheeses Ripened under Different Conditions. J. Food Sci..

[27] van den Oever S.P., Mayer H.K. (2021). Analytical assessment of the intensity of heat treatment of milk and dairy products. Int. Dairy J..

[28] Patel H.A., Singh H., Anema S.G., Creamer L.K. (2006). Effects of Heat and High Hydrostatic Pressure Treatments on Disulfide Bonding Interchanges among the Proteins in Skim Milk. J. Agric. Food Chem..

[29] Ma Y., Qing M., Zang J., Shan A., Zhang H., Chi Y., Chi Y., Gao X. (2022). Molecular interactions in the dry heat-facilitated hydrothermal gel formation of egg white protein. Food Res. Int..

[30] de Wit J.N. (2009). Thermal behaviour of bovine β-lactoglobulin at temperatures up to 150 °C. a review. Trends Food Sci. Technol..

[31] Xiong L., Li C., Boeren S., Vervoort J., Hettinga K. (2020). Effect of heat treatment on bacteriostatic activity and protein profile of bovine whey proteins. Food Res. Int..

[32] Van der Plancken I., Van Loey A., Hendrickx M.E.G. (2005). Changes in Sulfhydryl Content of Egg White Proteins Due to Heat and Pressure Treatment. J. Agric. Food Chem..

[33] Bonfatti V., Grigoletto L., Cecchinato A., Gallo L., Carnier P. (2008). Validation of a new reversed-phase high-performance liquid chromatography method for separation and quantification of bovine milk protein genetic variants. J. Chromatogr. A.

[34] Halabi A., Deglaire A., Hennetier M., Violleau F., Burel A., Bouhallab S., Dupont D., Croguennec T. (2020). Structural characterization of heat-induced protein aggregates in model infant milk formulas. Food Hydrocoll..

[35] Stănciuc N., Aprodu I., Râpeanu G., van der Plancken I., Bahrim G., Hendrickx M. (2013). Analysis of the Thermally Induced Structural Changes of Bovine Lactoferrin. J. Agric. Food Chem..

[36] Davis P.J., Williams S.C. (1998). Protein modification by thermal processing. Allergy.

[37] Wang L., Ma Y., Li H., Yang F., Cheng J. (2021). Identification and characterization of yak α-lactalbumin and β-lactoglobulin. J. Dairy Sci..

[38] Rogers D.M., Jasim S.B., Dyer N.T., Auvray F., Réfrégiers M., Hirst J.D. (2019). Electronic Circular Dichroism Spectroscopy of Proteins. Chem.

[39] Cristau P., Martin M.-T., Tran Huu Dau M.-E., Vors J.-P., Zhu J. (2004). Strained-Cyclophane-Induced β-Turn Template:  Design, Synthesis, and Spectroscopic Characterization. Org. Lett..

[40] Wang Q., Tolkach A., Kulozik U. (2006). Quantitative Assessment of Thermal Denaturation of Bovine α-Lactalbumin via Low-Intensity Ultrasound, HPLC, and DSC. J. Agric. Food Chem..

[41] Qie X., Chen W., Zeng M., Wang Z., Chen J., Goff H.D., He Z. (2021). Interaction between β-lactoglobulin and chlorogenic acid and its effect on antioxidant activity and thermal stability. Food Hydrocoll..

[42] Lee Y.H. (1992). Food-processing approaches to altering allergenic potential of milk-based formula. J. Pediatr..

[43] Huang Y., Cao H., Pan M., Wang C., Sun B., Ai N. (2024). Unraveling volatilomics profiles of milk products from diverse regions in China. Food Res. Int..

[44] Vagenas G., Roussis I.G. (2012). Fat-Derived Volatiles of Various Products of Cows’, Ewes’, and Goats’ Milk. Int. J. Food Prop..

[45] Perkins M., Elliott A., D’Arcy B., Deeth H. (2005). Stale flavour volatiles in Australian commercial UHT milk during storage. Aust. J. Dairy. Technol..

[46] Chi X., Shao Y., Pan M., Yang Q., Yang Y., Zhang X., Ai N., Sun B. (2021). Distinction of volatile flavor profiles in various skim milk products via HS-SPME–GC–MS and E-nose. Eur. Food Res. Technol..

[47] Goss M.B., Kroll J.H. (2024). Chamber studies of OH + dimethyl sulfoxide and dimethyl disulfide: Insights into the dimethyl sulfide oxidation mechanism. Atmos. Chem. Phys..

[48] Valero E., Villamiel M., Miralles B., Sanz J., Martínez-Castro I. (2001). Changes in flavour and volatile components during storage of whole and skimmed UHT milk. Food Chem..

[49] Melini F., Melini V., Luziatelli F., Ruzzi M. (2017). Raw and Heat-Treated Milk: From Public Health Risks to Nutritional Quality. Beverages.

[50] Kalač P. (2011). The effects of silage feeding on some sensory and health attributes of cow’s milk: A review. Food Chem..

[51] Yayota M., Tsukamoto M., Yamada Y., Ohtani S. (2013). Milk composition and flavor under different feeding systems: A survey of dairy farms. J. Dairy Sci..

[52] Cornu A., Rabiau N., Kondjoyan N., Verdier-Metz I., Pradel P., Tournayre P., Berdagué J.L., Martin B. (2009). Odour-active compound profiles in Cantal-type cheese: Effect of cow diet, milk pasteurization and cheese ripening. Int. Dairy J..

[53] Di C., Jia W. (2023). Capture modes evaluation of four flavor constituents in β-lactoglobulin by high-resolution mass spectrometry and molecular dynamics simulation approaches. Food Hydrocoll..

