# Supplementary material for: Effect of Whey Protein Changes on Milk Flavor and Sensory Characteristics During Heating
Source: Foods. 2024 Dec 26;14(1):33. doi: 10.3390/foods14010033 (PMC11720481; doi:10.3390/foods14010033)
Supplement: Supplementary file 1 [file foods-14-00033-s001.zip › Supplementary Materials.pdf]

Supplementary Materials

Table S1: The list of milk attributes in the Quantitative Description Analysis.

Descriptors	Definition	References
Milky aroma	normal and natural milk aroma, no other off-flavors	whole milk
Dairy fat	the oily aromatics reminiscent of milk or dairy fat	whipping cream
Cooked	sulfurous, cooked cabbage flavor	heat the milk at 80°C for 1 minute
Oxidized	burnt feathers flavor	milk exposed to light for 18 h
Grassy	the aromatics associated with lawns after it's been mowed	hexanal

Table S2: The identification and quantification of the VOCs in the milk exposed to different heat treatments using HS-SPME-Arrow-GC-MS.

No.	Compound	Odor description [a]	CAS	Content (μg/kg)							RI	NIST RI	Identification [b]
				Raw	6010	6030	7510	7530	10010	10030			
	Alcohols												
1	1-Butanol	medicine, fruit, wine	71-36-3	1.79±0.26	1.98±0.25	1.58±0.18	2.23±0.09	2.55±0.04	0.74±0.13	1.44±0.30	1135	1150	MS, RI
2	2-Methyl-1-butanol	malt, wine, onion	137-32-6	0.39±0.06	1.75±0.61	0.56±0.15	0.65±0.06	7.68±0.07	0.09±0.02	1.38±0.02	1206	1207	MS, RI
3	1-Pentanol	balsamic	71-41-0	0.30±0.06	0.35±0.04	0.23±0.05	0.33±0.03	0.73±0.05	0.13±0.02	0.39±0.03	1263	1255	MS, RI
4	3-Methyl-1-pentanol		589-35-5	0.33±0.03	0.31±0.01	0.33±0.02	0.16±0.02	0.58±0.06	0.20±0.01	0.40±0.02	1321	1320	MS, RI
5	1-Hexanol	resin, flower, green	111-27-3	1.25±0.05	1.13±0.13	1.34±0.05	0.88±0.01	0.88±0.04	0.78±0.00	1.27±0.05	1353	1359	MS, RI
6	1-Octen-3-ol	mushroom	3391-86-4	0.70±0.03	0.59±0.06	0.70±0.01	0.34±0.04	0.39±0.03	0.37±0.02	0.50±0.01	1448	1451	MS, RI
7	2-Ethyl-1-hexanol	rose, green	104-76-7	1.38±0.11	1.35±0.30	1.07±0.04	1.62±0.20	1.66±0.16	1.28±0.08	1.83±0.40	1486	1484	MS, RI
8	1-Octanol	chemical, metal, burnt	111-87-5	0.55±0.05	0.81±0.09	1.12±0.08	0.77±0.09	0.94±0.13	0.54±0.06	0.81±0.04	1558	1456	MS, RI
9	1-Dodecanol	fat, wax	112-53-8	0.03±0.00	0.07±0.01	0.09±0.02	0.30±0.04	0.36±0.03	0.29±0.05	0.75±0.04	1967	1981	MS, RI
				6.72	8.34	7.02	7.28	15.77	4.42	8.77			
	Aldehydes												
10	Hexanal	grass, tallow, fat	66-25-1	0.07±0.00	0.11±0.02	0.10±0.00	0.93±0.20	1.40±0.14	1.69±0.06	1.85±0.06	1087	1079	MS, RI
11	Octanal	fat, soap, lemon, green	124-13-0	0.24±0.02	0.73±0.13	0.43±0.07	0.76±0.15	0.33±0.09	0.63±0.33	1.42±0.06	1294	1287	MS, RI
12	Nonanal	fat, citrus, green	124-19-6	1.30±0.00	2.48±0.39	2.71±0.03	3.60±0.45	0.87±0.04	3.01±0.25	5.09±0.80	1400	1390	MS, RI
13	(E)-2-Octenal	green, nut, fat.	2548-87-0	0.32±0.02	0.34±0.01	0.41±0.02	0.49±0.03	0.42±0.09	0.47±0.07	0.95±0.12	1435	1434	MS, RI
14	Benzaldehyde	almond, burnt sugar	100-52-7	0.79±0.06	0.69±0.11	0.68±0.13	2.95±0.33	7.82±0.57	9.52±1.01	17.29±0.87	1537	1528	MS, RI
15	(E)-2-Nonenal	cucumber, fat, green	18829-56-6	0.04±0.01	0.09±0.01	0.10±0.03	0.23±0.03	0.15±0.01	0.22±0.02	0.38±0.03	1543	1542	MS, RI
				2.76	4.44	4.43	8.96	10.99	15.54	26.98			
	Acids												
16	Acetic acid	sour	64-19-7	2.06±0.00	1.92±0.05	1.19±0.02	15.09±0.11	39.15±0.21	11.81±0.32	17.00±0.62	1469	1461	MS, RI

17	Propanoic acid	pungent, rancid, soy	79-09-4	0.38±0.10	0.26±0.03	0.21±0.02	0.80±0.05	1.19±0.04	0.59±0.13	0.80±0.14	1549	1540	MS, RI
18	Butyric acid	rancid, cheese, sweat	107-92-6	77.50±0.88	9.52±0.08	6.48±0.03	21.53±3.68	18.26±0.14	14.62±1.98	20.85±0.62	1639	1631	MS, RI
19	Isovaleric acid	sweat, acid, rancid	503-74-2	-	0.05±0.01	-	0.12±0.05	0.24±0.01	0.18±0.03	0.21±0.04	1679	1687	MS, RI
20	Pentanoic acid	sweat	109-52-4	2.95±0.02	0.62±0.15	0.54±0.13	1.43±0.19	1.70±0.50	1.01±0.15	1.74±0.05	1742	1743	MS, RI
21	Hexanoic acid	sweat	142-62-1	347.68±5.95	37.41±0.35	30.92±0.19	64.14±0.69	70.16±0.45	48.85±0.85	59.83±0.89	1852	1852	MS, RI
22	2-Ethyl-1-hexanoic acid		149-57-5	0.27±0.03	2.88±0.41	1.71±0.33	5.51±0.46	9.89±0.96	8.79±1.16	26.59±1.16	1956	1954	MS, RI
23	Heptanoic acid		111-14-8	6.60±0.08	0.91±0.18	0.85±0.24	2.57±0.36	3.27±0.78	2.24±0.37	3.72±0.22	1959	1967	MS, RI
24	Octanoic acid	Sweat, cheese	124-07-2	27.48±0.08	4.32±0.77	4.47±1.59	12.72±1.51	17.40±2.49	13.71±1.54	16.79±1.06	2064	2070	MS, RI
25	Nonanoic acid	green, fat	112-05-0	1.12±0.02	0.34±0.05	0.41±0.17	1.61±0.17	2.59±0.39	1.68±0.14	2.86±0.00	2171	2169	MS, RI
26	Decanoic acid	rancid, fat	334-48-5	42.16±0.11	9.86±0.09	8.68±0.31	47.21±4.95	68.79±0.29	48.12±0.59	70.69±0.46	2277	2273	MS, RI
27	9-Decenoic acid		14436-32-9	0.92±0.04	0.18±0.02	0.28±0.01	0.99±0.12	1.73±0.30	1.28±0.39	1.46±0.06	2340	2348	MS, RI
28	Undecanoic acid	sweat	112-37-8	0.24±0.03	0.06±0.00	-	0.46±0.05	0.87±0.19	0.75±0.06	0.96±0.11	2374	2365	MS, RI
29	Dodecanoic acid	metal	143-07-7	3.59±0.23	1.14±0.07	1.06±0.04	8.32±1.38	12.42±0.06	6.14±0.17	14.46±0.09	2480	2469	MS, RI
30	Tetradecanoic acid		544-63-8	1.57±0.11	1.04±0.10	1.27±0.03	11.94±0.18	13.46±0.23	5.89±0.04	17.58±0.35	2694	2689	MS, RI
				514.52	70.51	58.07	194.44	261.12	165.66	255.54			
	Esters												
31	Butyl acetate	pear	123-86-4	0.32±0.02	0.43±0.04	0.43±0.01	0.50±0.06	0.49±0.02	4.10±0.29	0.36±0.04	1074	1066	MS, RI
32	Pentyl formate		638-49-3	2.59±0.04	1.70±0.23	1.82±0.02	1.39±0.11	1.36±0.05	2.74±0.16	1.19±0.14	1122	1187	MS, RI
33	Hexyl acetate	fruit, herb	142-92-7	0.05±0.01	0.38±0.04	0.13±0.00	0.44±0.03	0.17±0.02	0.08±0.02	0.41±0.03	1275	1276	MS, RI
34	γ-Butyrolactone	caramel, sweet	96-48-0	-	-	0.07±0.02	-	6.48±0.02	0.74±0.21	1.02±0.32	1646	1643	MS, RI
35	2(5H)-Furanone		497-23-4	-	-	0.52±0.07	0.75±0.10	7.56±0.10	1.76±0.82	3.08±0.12	1773	1767	MS, RI
36	δ-Hexalactone		823-22-3	0.87±0.09	0.92±0.08	1.30±0.22	1.36±0.12	2.28±0.29	2.84±0.40	3.08±0.03	1806	1792	MS, RI
37	6-Propyltetrahydro-2H-pyran-2-one	peach	698-76-0	2.31±0.21	1.98±0.15	2.67±0.49	5.03±0.81	7.68±0.10	7.46±0.51	10.29±1.04	1982	1985	MS, RI
38	γ-Nonalactone	coconut, peach	104-61-0	0.08±0.01	0.06±0.00	0.10±0.05	0.50±0.04	0.57±0.02	0.38±0.06	0.71±0.06	2043	2037	MS, RI
39	6-Pentyltetrahydro-2H-pyran-2-one	coconut	705-86-2	7.76±0.48	5.04±0.42	6.14±0.26	23.85±0.07	34.26±0.25	33.74±0.28	50.96±0.25	2208	2210	MS, RI
40	Methyl palmitate		112-39-0	-	-	-	0.14±0.02	0.19±0.03	0.23±0.18	0.23±0.05	2212	2213	MS, RI
41	δ-Dodecalactone	fruit, sweet	713-95-1	1.74±0.09	1.09±0.01	1.46±0.13	7.40±0.86	10.52±0.08	8.46±0.04	22.19±0.36	2441	2458	MS, RI

				15.72	11.6	14.64	41.36	71.568	62.53	93.52			
	Ketones											
42	2,3-Butanedione	butter	431-03-8	0.84±0.01	0.24±0.02	0.91±0.04	0.95±0.02	0.09±0.00	0.71±0.18	1.84±0.22	978	971	MS, RI
43	3-Hexanone	ether, grape	589-38-8	3.29±0.11	1.23±0.17	0.76±0.03	3.97±0.21	3.93±0.16	0.84±0.04	3.97±0.12	1076	1050	MS, RI
44	5-Methyl-2-hexanone		110-12-3	9.53±0.30	3.87±0.10	12.01±0.66	2.51±0.21	1.54±0.01	11.67±0.51	21.73±0.38	1145	1155	MS, RI
45	2-Heptanone	soap	110-43-0	1.05±0.02	1.12±0.02	1.40±0.05	5.58±0.08	7.76±0.47	31.48±4.29	69.64±0.62	1183	1184	MS, RI
46	4-Octanone		589-63-9	0.33±0.03	0.43±0.04	0.38±0.06	0.30±0.03	0.48±0.07	0.36±0.04	0.33±0.08	1226	1224	MS, RI
47	2-Nonanone	hot milk, soap, green	821-55-6	0.28±0.00	0.20±0.02	0.43±0.17	0.68±0.07	1.73±0.19	6.32±0.61	13.10±0.91	1393	1389	MS, RI
48	2-Undecanone	orange, fresh, green	112-12-9	0.52±0.01	0.50±0.05	0.71±0.00	2.26±0.31	5.10±1.05	17.76±0.32	40.22±3.80	1603	1596	MS, RI
49	Acetophenone	must, flower, almond	98-86-2	0.99±0.03	1.04±0.18	0.94±0.06	2.03±0.17	2.19±0.30	1.81±0.18	2.02±0.06	1662	1669	MS, RI
50	2-Tridecanone		593-08-8	0.08±0.02	0.09±0.02	0.16±0.02	0.85±0.03	2.28±0.79	3.72±0.14	14.62±0.22	1810	1816	MS, RI
51	2-Pentadecanone	Fresh, jasmine	2345-28-0	0.04±0.01	-	0.39±0.02	1.17±0.07	1.46±0.04	1.52±0.26	11.49±0.08	2020	2028	MS, RI
				16.95	8.72	18.09	20.30	26.56	76.19	178.96			
	Others											
52	Styrene	balsamic, gasoline	100-42-5	1.15±0.03	1.16±0.17	1.02±0.16	1.52±0.18	-	1.54±0.19	1.52±0.12	1261	1259	MS, RI
53	Dimethyl sulfoxide		67-68-5	0.27±0.01	0.23±0.05	0.20±0.07	0.49±0.11	0.81±0.33	0.53±0.09	0.57±0.05	1584	1579	MS, RI
54	(E)-2-Octenol	soap, plastic	18409-17-1	0.09±0.01	0.08±0.00	0.08±0.01	-	-	-	3.97±0.39	1614	1622	MS, RI
55	3-Furanmethanol		4412-91-3	-	-	0.75±0.04	-	-	7.00±0.10	8.53±0.30	1680	1679	MS, RI
56	Dimethyl sulfone	sulfur, burnt	67-71-0	4.85±0.22	4.27±0.72	3.88±0.10	7.20±0.15	8.99±0.03	5.94±0.71	6.40±0.37	1912	1912	MS, RI
57	Phenol	phenol	108-95-2	0.23±0.02	0.23±0.04	0.22±0.02	0.52±0.08	0.71±0.12	0.47±0.07	0.59±0.07	2015	2015	MS, RI
58	2,5-Dimethyl-4-hydroxy-3(2H)-furanone	caramel	3658-77-3	-	-	-	-	1.71±0.06	0.21±0.06	0.47±0.03	2040	2039	MS, RI
59	2,4-Di-tert-butylphenol		96-76-4	3.59±0.16	4.05±0.43	3.99±0.07	9.96±0.99	11.66±1.60	5.04±0.03	9.42±1.17	2316	2321	MS, RI
				10.18	10.02	10.14	19.69	23.88	20.73	31.47			

[a] The odor descriptions of the compounds were matched via https://www.flavornet.org/flavornet.html.
[b] The VOCs were identified via mass spectrometry (MS) and the retention index method (RI).
-: The compound was not detected.
